# Relevance of STK11 Mutations Regarding Immune Cell Infiltration, Drug Sensitivity, and Cellular Processes in Lung Adenocarcinoma

**DOI:** 10.3389/fonc.2020.580027

**Published:** 2020-12-23

**Authors:** Zhenqing Li, Bo Ding, Jianxun Xu, Kai Mao, Pengfei Zhang, Qun Xue

**Affiliations:** ^1^ Research Center of Clinical Medicine, Affiliated Hospital of Nantong University, Nantong, China; ^2^ Medical College of Nantong University, Nantong, China; ^3^ Cardiovascular Surgery Department, Affiliated Hospital of Nantong University, Nantong, China

**Keywords:** lung adenocarcinoma, STK11 mutation, TCGA, RNA sequencing, bioinformatics analysis

## Abstract

Serine/threonine kinase 11 (STK11) is one member of the serine/threonine kinase family, which is involved in regulating cell polarity, apoptosis, and DNA damage repair. In lung adenocarcinoma (LUAD), it can play as one tumor suppressor and always be mutated. In this study, we aimed to assess the relevance of STK11 mutations in LUAD, in which we also studied the correlation among immune cell infiltration, drug sensitivity, and cellular processes. By performing the bioinformatics analysis of the Cancer Genome Atlas (TCGA) about LUAD patients, we found that the mutation efficiency of STK11 mutations is about 19%. Additionally, the differentially expressed gene analysis showed that there were 746 differentially expressed genes (DEGs) between LUAD patients with and without STK11 mutations. Kyoto Encyclopedia of Genes and Genomes (KEGG) and Gene Ontology (GO) analysis showed that the DEGs were enriched in various tumorigenesis signaling pathways and metabolic processes. Among these DEGs, the top ranking 21 genes were found that they were more frequently mutated in the STK11 mutation group than in the wild-type group (p-value<0.01). Finally, the LUAD patients with STK11 mutations suffered the worse immune cell infiltration levels than the LUAD patients with wild-type. The STK11 gene copy number was correlated with immune cell infiltration. Aiming to develop the therapeutic drugs, we performed Genomics of Drug Sensitivity in Cancer (GDSC) data to identify the potential therapeutic candidate and the results showed that Nutlin-3a(-) may be a sensitive drug for LUAD cases harboring STK11 mutations. The specific genes and pathways shown to be associated with LUAD cases involving STK11 mutations may serve as targets for individualized LUAD treatment.

## Introduction

Lung cancer is the most common cancer, which accounts for 11.6% of cases in all types of cancer cases and causes the 18.4% of cancer-related deaths (1). Classified by clinicopathological features, lung cancers are defined as small cell lung cancers (13%) or non-small cell lung cancers (NSCLC; 87%), which have distinct therapeutic implications. The NSCLC is the main subtype of lung adenocarcinoma (LUAD). While NSCLC patients suffered the low 5-year overall survival rate of 18.2% (2), but targeted molecular therapies have considerably improved the survival of NSCLC patients in the past decade. These excellent therapeutic status always suffered with epidermal growth factor receptor (EGFR) mutations, anaplastic lymphoma kinase (ALK), c-ros proto-oncogene 1 (ROS1), or ret proto-oncogene (RET) translocations, or other activated oncogenes (3, 4). Therefore, it is important to identify latent biomarkers of NSCLC progression and prognosis that represent potential targets for developing targeted molecular therapy.

Recently, serine/threonine kinase 11 (STK11) has been identified as a tumor suppressor gene, which always is silenced in the wide spectrum of truncating mutations. STK11 mutation cannot be able to inhibit mammalian target of rapamycin (mTOR) (5–7). In contrast, it has been reported that a short STK11 isoform with absence of 124 N-terminal amino acids is an oncogene (8). However, it is known as the tumor promotion mechanisms of tumor protein P53 (TP53).

Herein, aiming to understand the relevance of STK11 mutations with LUAD survival, we systematically studied the survival of LUAD patients with or without harboring STK11 mutation. Then, we identified the differentially expressed genes (DEGs) and conducted the GSEA enrichment analysis on the DEGs to explore the enriched cellular processes in the STK11 mutation group. Moreover, we also assessed the correlation between STK11 mutation and immune cell infiltration by using the Genomics of Drug Sensitivity in Cancer (GDSC) database to identify a candidate drug to treat LUAD with STK11 mutations. By this investigation, STK11 may serve as a target for the individualized and precise therapy of LUAD.

## Materials and Methods

### RNA-seq Data

We obtained RNA-seq data and relative clinical data (including survival and progression status, STK11 mutation type, and STK11 copy number) on LUAD patients with STK11 mutations *via* the “cBioPortal for Cancer Genomics” website (9). This involved data on 561 LUAD patients (including RNA-seq profiles) from The Cancer Genome Atlas (TCGA) database. These patients were divided into an STK11 mutation group and a wild-type group.

### Drug Sensitivity Analysis Using the GDSC Database

We utilized the GDSC database to identify potential therapeutic compounds that were sensitive for LUAD cases harboring STK11 mutations. We then constructed a volcano plot, an elastic network and a scatter diagram. Finally, we carried out a Mann–Whitney–Wilcoxon (MWW) analysis to explore the potential applications.

### Identification of Differentially Expressed Genes

We used the EdgeR package to identify DEGs between LUAD patients with or without STK11 mutations (10, 11). The criteria for defining DEGs were as follows: p-value <0.05, false discovery rate (FDR) q-value <0.25, and |log2(fold change)| ≥1. The DEGs were then subjected to bioinformatics analysis.

### Kyoto Encyclopedia of Genes and Genomes and Gene Ontology Analyses of the DEGs

GO and KEGG analyses were carried out using the clusterProfiler package in R to automatically determine the enriched categories and pathways for each gene cluster, and to visualize the data. To avoid a high FDR due to multiple comparisons, q-values were calculated. FDR-adjusted q-value <0.25 and p-value <0.05 were considered as statistically significant.

### Gene Set Enrichment Analysis of the DEGs

We used GSEA software version 3.0 to conduct GO and KEGG analysis of the DEGs that were differentially regulated between the LUAD patients with or without STK11 mutations. Enrichment results with an FDR-adjusted q-value <0.25 and a p-value <0.05 were considered as statistically significant.

### Protein–Protein Interaction Network and Module Analysis

We used the Search Tool for the Retrieval of Interacting Genes (STRING; http://string-db.org) to create a PPI network of co-regulated hub genes and assess significant mutual effects among these proteins encoded by the DEGs (12). We then used Cytoscape (v3.0, https://cytoscape.org/) software to identify and visualize the mutual impact relationships among these proteins. Interactions with a confidence score >0.4 were selected for use in the PPI network. Using the Molecular Complex Detection (MCODE) plug-in in Cytoscape, we identified the PPI modules. In addition, we carried out KEGG and GO enrichment analyses of the DEGs in the top four modules.

### KEGG Pathway Enrichment Analysis of Genes with Differential Mutation Rates

Next, we identified the genes that were more frequently mutated in the STK11 mutation group than the wild-type group. A KEGG analysis was carried out using the clusterProfiler package to automatically determine the enriched pathways for each gene cluster and to visualize the data. To avoid a high FDR due to multiple comparisons, q-values were calculated. FDR-adjusted q-value <0.25 and p-value <0.05 were considered statistically significant.

### Correlation of STK11 Mutation With Immune Cell Infiltration

Using Tumor Immune Estimation Resource (TIMER) data, we identified the correlations between STK11 mutation and immune cell infiltration, STK11 copy number and immune cell infiltration, STK11 mutation and STK11 mRNA expression, STK11 copy number/mutation type and STK11 mRNA expression, and STK11 mRNA expression and immune cell infiltration, based on Pearson’s correlation coefficients and the associated p-values.

### Statistical Analyses

GraphPad and R version 3.3.0 were used for the statistical analyses. The DEG identification analysis and GO and KEGG enrichment analyses (including the GSEA enrichment analyses) were adjusted for multiple comparisons using the Benjamini–Hochberg procedure. The results were considered statistically significant based on p-value <0.05 and FDR-adjusted q-value <0.25.

## Results

### Clinical Information and Basic Data of LUAD Harboring STK11 Mutations

**Figure 1:**
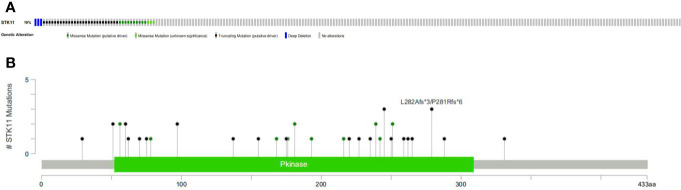
Frequency **(A)** and types **(B)** of STK11 mutations in lung adenocarcinoma (LUAD) patients.

RNA-seq and clinical data (including survival time and ratio, STK11 mutation type and STK11 copy number) on 561 LUAD patients (with complete follow-up data) were downloaded from the TCGA database. Of these patients, 19% had STK11 mutations (**Figure 1A**). Mutation types of STK11 gene included truncating mutations, deep deletions and missense mutations (**Figure 1B**). These mutation information indicated by the TCGA data obtained *via* the “cBioPortal for Cancer Genomics” website. Further to explore the correlation between STK11 and clinicopathological characters, we found that the STK11 status is highly associated with the LUAD grade (as shown in **Table 1**).

**Table 1:** The correlation between clinicopathological characters.

Terms	HR	HR.95L	HR.95H	p value
age	1.001004	0.983617	1.018699	0.910589
gender	1.073169	0.765986	1.503543	0.681479
stage	1.585823	1.35545	1.855351	8.54E-09
T	1.603003	1.318279	1.949221	2.25E-06
M	1.859276	1.047497	3.300161	0.034135
N	1.734613	1.429159	2.105352	2.50E-08
STK11	1.493044	0.96699	2.305279	0.070528

### STK11 Mutation and Immune Cell Infiltration

**Figure 2:**
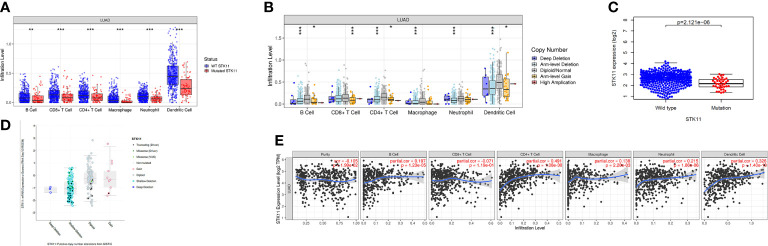
Correlations between STK11 mutations and immune cell infiltration/STK11 mRNA expression. Correlations between **(A)** STK11 mutation and immune cell infiltration, **(B)** STK11 copy number and immune cell infiltration, **(C)** STK11 mutation and STK11 mRNA expression, **(D)** STK11 copy number/mutation type and STK11 mRNA expression, and **(E)** STK11 mRNA expression and immune cell infiltration.

Tumor environment has a great impact on patient survival, and the immune cell infiltration indicated the complex environment. Here, we explored the differences in immune cell infiltration between patients with and without STK11 mutations. The analysis showed that the LUAD patients with STK11 mutations had fewer infiltrating immune cells, including B cells (p-value < 0.01), CD8+ T cells (p-value < 0.001), CD4+ T cells (p-value < 0.001), macrophages (p-value < 0.001), neutrophils (p-value < 0.001), and dendritic cells (p-value < 0.001) (**Figure 2A**). For dendritic cell infiltration, the infiltration level is significantly decreased to less than 0.3. Additionally, the STK11 copy number was correlated with immune cell infiltration, in which abnormal copy number of STK11 mutation also caused the fewer infiltrating immune cells (**Figure 2B**). Comparison of the STK11 mutation group with the wild-type group showed the STK11 mRNA expression was significantly downregulated (**Figure 2C**). By identifying the different mutation types, the STK11 mutation type and copy number were positively correlated with STK11 mRNA expression (**Figure 2D**). However, the STK11 mRNA expression was not very strongly correlated with immune cell infiltration (**Figure 2E**), except the T cell CD4+ and dendritic cell.

### STK11 Mutation and Drug Sensitivity

**Figure 3:**
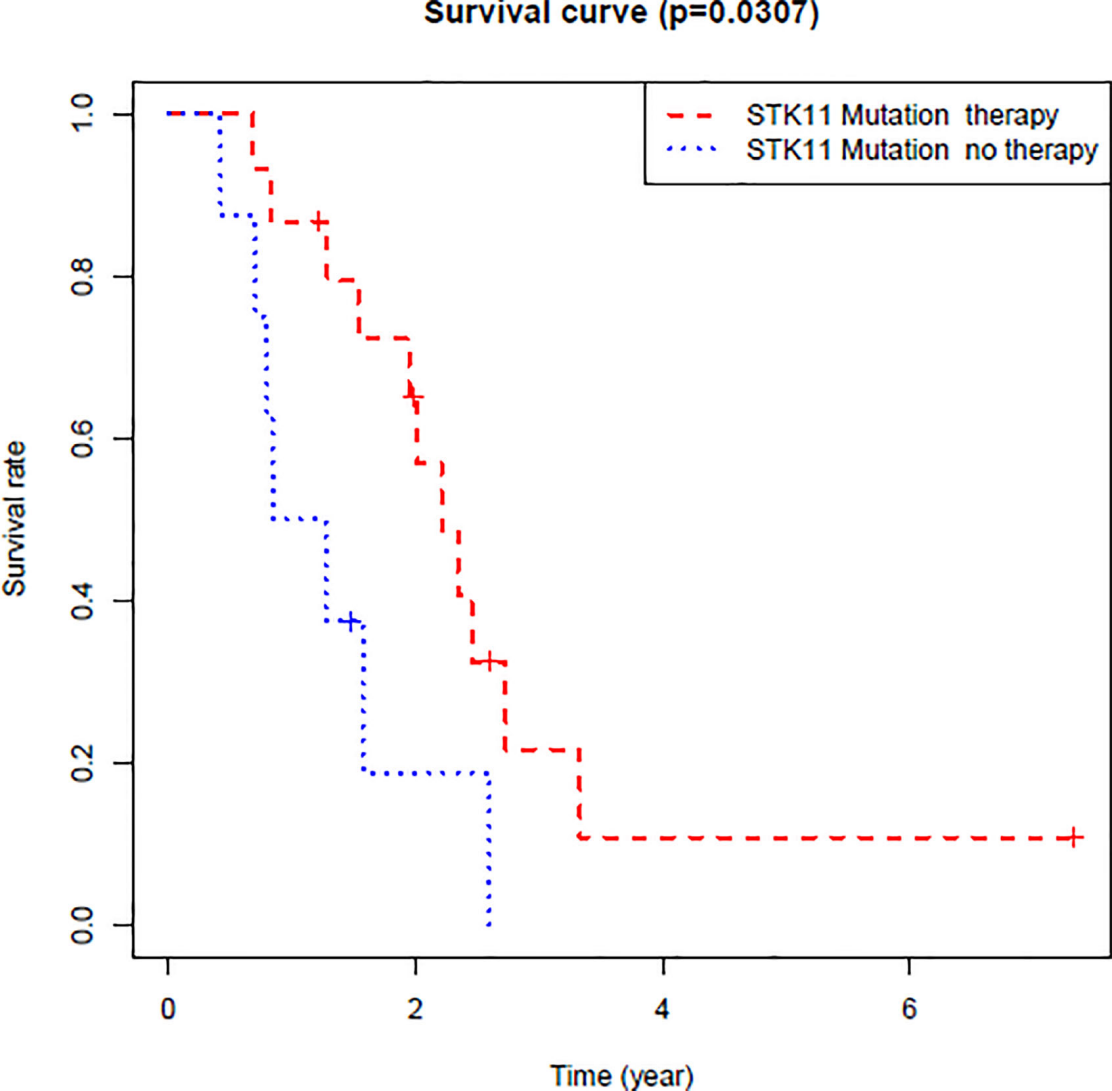
Overall survival of LUAD patients with or without clinical interventions. These LUAD patients harbored STK11 mutation.

**Figure 4:**
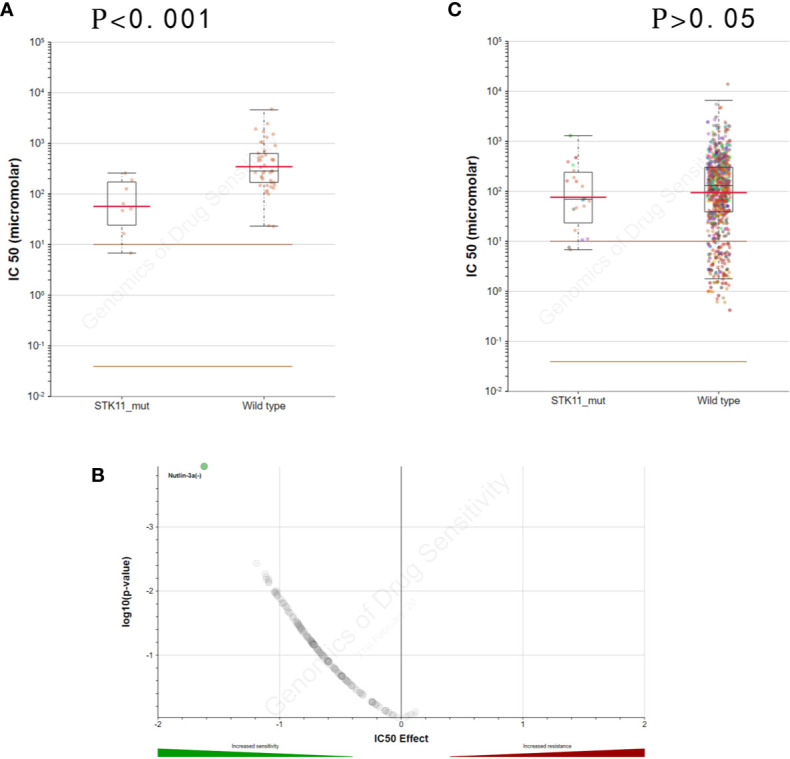
STK11 mutation and drug sensitivity. **(A, B)** Genomics of Drug Sensitivity in Cancer (GDSC) database results showing that lung adenocarcinoma (LUAD) cells with STK11 mutations (other cancer types were excluded) are inhibited by Nutlin-3a(-) (p-value < 0.001). **(C)** Scatter and volcano plots showing that multiple other cancer cell types with STK11 mutations are not inhibited by Nutlin-3a(-) (p-value > 0.05).

By exploring the correlation between STK11 status and clinical interventions, we found that LUAD patients harboring the STK11 mutation can obtain better overall survival (longrank p = 0.0307) if patients accepted the clinical interventions (**Figure 3**). Consequently, we further explored the role of STK11 mutation status in LUAD treatment. To identify the potential effect of anti-tumor drugs, we used the GDSC database to attempt to identify potentially sensitive and selective drugs for patients with or without STK11 mutation. The GDSC screening results revealed that Nutlin-3a(-) may exhibit sensitivity for LUAD with STK11 mutations (**Figures 4A, B**), while not exhibiting sensitivity to other cancer types with STK11 mutations (**Figure 4C**). This may make it a useful targeted drug therapy for LUAD patients with STK11 mutations.

### GSEA Enrichment Analysis of DEGs

**Figure 5:**
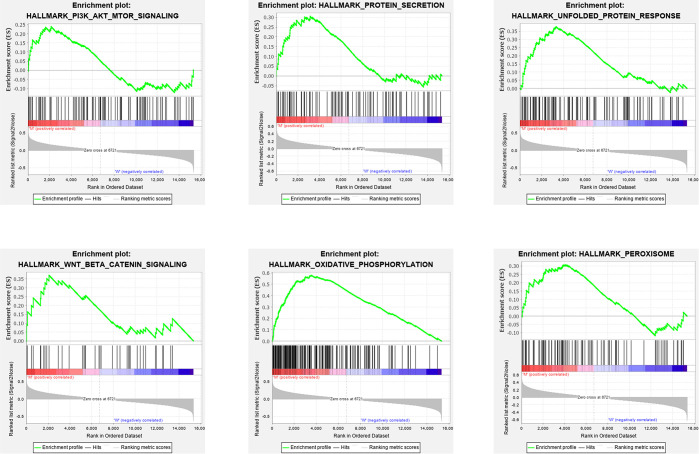
Gene set enrichment analysis (GSEA) GO and KEGG enrichment results for the differentially expressed genes (DEGs) that were differentially regulated between the lung adenocarcinoma (LUAD) patients with or without STK11 mutations.

The clinical information analysis and related survival correlation analysis reveal that STK11 mutations may play a key role in the progression and prognosis of LUAD. To consider that cellular genome can determined the clinical intervention approaches, we further investigated the effects of STK11 mutations on the cellular signaling processes in LUAD. Using GSEA analysis, the results showed that the functional gene sets are strongly related to STK11 mutation status. The following GO and KEGG categories were markedly enriched: oxidative phosphorylation, unfolded protein response, peroxisome, protein secretion, Wnt/beta catenin signaling, and PI3K/AKT/mTOR signaling (**Figure 5**).

### DEGs Between LUAD Patients With and Without STK11 Mutations

**Figure 6:**
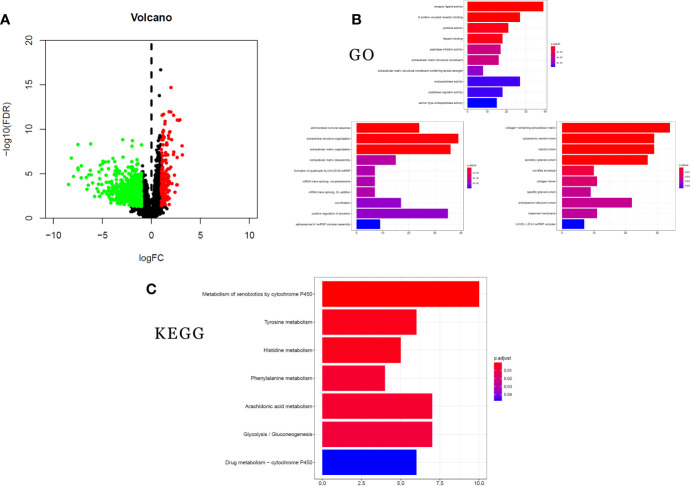
clusterProfiler enrichment results for the differentially expressed genes (DEGs). **(A)** Volcano plot of DEGs. **(B)** Gene Ontology (GO) enrichment analysis of DEGs. **(C)** Kyoto Encyclopedia of Genes and Genomes (KEGG) pathway enrichment analysis of DEGs.

To analyze the altered gene expression and pathways related to STK11 mutations in LUAD, 746 DEGs (|log2(FoldChange)| ≥1.0 and p-value <0.05) were identified using the RNA-seq data. Among these DEGs (**Figure 6A**), 172 genes were upregulated and 574 genes were downregulated. The higher inhibition of critical genes indicated that STK11 mutation may silence some specific signaling to worsen the patients’ survival.

### GO and KEGG Analyses of the DEGs

GO and KEGG analysis can provide more information about the specific signaling pathways affected by STK11 mutation. To study the functions of the 746 DEGs, we used the clusterProfiler package to analyze GO annotations and KEGG pathways (FDR-adjusted q-value <0.25 and p-value <0.05 were considered as statistically significant). GO analysis of the DEGs showed top five ranking enrichment regarding the following terms: extracellular matrix disassembly, cytokine activity, G protein-coupled receptor binding, receptor ligand activity and cytoplasmic vesicle lumen (**Figure 6B**). Meanwhile, the KEGG pathway analysis revealed significant enrichment regarding the following pathways: arachidonic acid metabolism, phenylalanine metabolism, histidine metabolism, and tyrosine metabolism (**Figure 6C**). The significant enrichment of metabolic pathways revealed that STK11 mutation mainly affected the metabolic approaches, and it is consistent with GO enrichment results.

### PPI Network and Module Analysis

**Figure 7:**
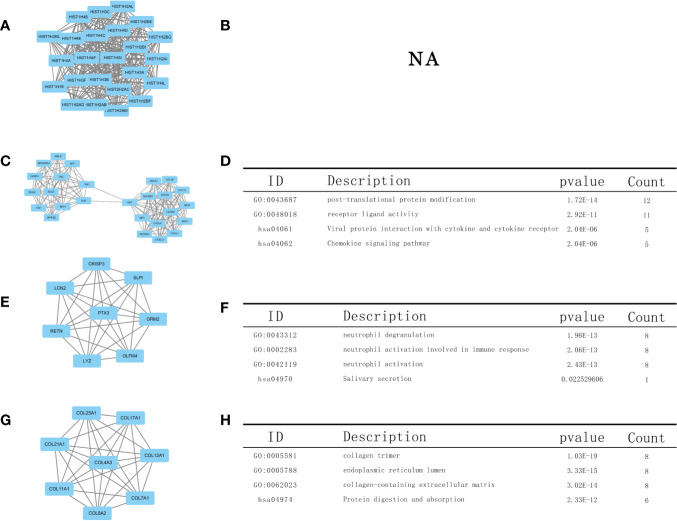
Top four modules in the protein–protein interaction (PPI) network. PPI network and Gene Ontology (GO) and Kyoto Encyclopedia of Genes and Genomes (KEGG) analyses of modules **(A, B)** 1, **(C, D)** 2, **(E, F)** 3, and **(G, H)** 4.

Using the STRING database, we constructed a PPI network, exploring the mutual effects and hub DEGs. We identified the PPI modules using the MCODE plug-in in Cytoscape and conducted GO and KEGG analyses for the top four modules and parsed the annotation of relevant genes (**Figure 7**). The KEGG and GO analyses of the first module were abandoned because the results were NA (there is no result). However, the enrichment results revealed that DEGs in modules 2–4 were linked with the following terms: chemokine signaling pathway, neutrophil activation involved in immune response, neutrophil activation, receptor ligand activity, and protein digestion and absorption.

### KEGG Analysis of Mutated Genes in the STK11 Mutation Group

**Figure 8:**
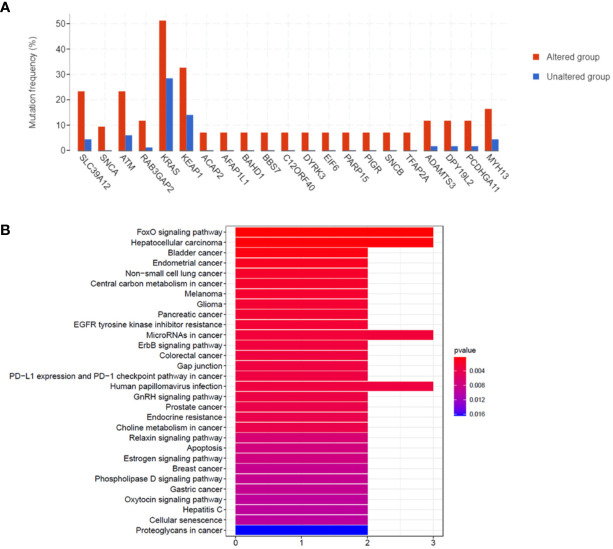
**(A)** Comparisons of the mutation frequencies of genes between the STK11 mutation and wild-type groups of lung adenocarcinoma (LUAD) patients (p-value < 0.01). **(B)** KEGG analysis of the genes with differential mutation rates.

There were 21 genes that were more frequently mutated in the STK11 mutation group than the wild-type group (p-value < 0.01) (**Figure 8A**). The KEGG analysis indicated enrichment regarding the FoxO signaling pathway, central carbon metabolism in cancer, NSCLC, ErbB signaling pathway, microRNAs in cancer and gap junction (**Figure 8B**).

## Discussion

The human STK11 gene, which also can be called as LKB gene, directly encodes the serine/threonine protein kinase. The major biological functions of STK11 are performed by binding with AMPK1 protein and further regulating the mTORC1 proteins. The mTOR signal pathway can regulate cell proliferations. In addition to its role in 5′ AMP-activated protein kinase (AMPK)-regulated energy metabolism, it is also involved in various cellular processes, including the regulation of cell cycle arrest (13), p53-mediated apoptosis (14), Wnt signaling (15, 16), transforming growth factor (TGF)-*β* signaling (17), RAS-induced cell transformation (18), and cell polarity (19). Although the role of STK11 in PI3K/RAS/mTORC1 signaling pathway has been well investigated, the specific mechanism of the tumor suppressor gene STK11 is not completely well-understood. Here, we assessed the relevance of STK11 mutations regarding immune cell infiltration, drug sensitivity, and cellular processes, which can provide insights for the development of individualized cancer treatments. By performing the clinical information analysis, we found that 19% of LUAD patients harbored the STK11 mutations. Among these mutations, three types of mutation can be identified, *i.e.* truncating mutations, deep deletions and missense mutations (**Figure 1B**).

To our knowledge, the metastasis of LUAD is the most lethal results, especially for brain metastasis. Many works have reported that the immune microenvironment can significantly affect the metastasis of LUAD cancer cells. As a result, to explore the immune cell infiltration can be provide more information about the critical factors to affect the survival of LUAD patients. Using the TIMER database, we found that the STK11 mutation group had induce the less immune cell infiltration, including B cells, CD8+ T cells, CD4+ T cells, macrophages and dendritic cells (**Figures 2A, B**). It meant that LUAD harboring STK11 mutations can cause the “cold” tumor immune microenvironment, which is owing to the low expression of STK11 genes (**Figures 2C, D**). Moreover, we only observed that the T cell CD4+ and dendritic cell are highly and positively associated with the STK11 expression level, while dendritic cell and T cell CD4+ are the common immunosuppressive immune cells. These results confirmed that mutation of STK11 can cause “cold” tumor immune microenvironment.

After identifying the relationship between STK11 mutation and LUAD immune microenvironment, to provide the potential therapeutic drugs may be necessary. By further analysis of the TCGA database of LUAD patients harboring STK11 mutation, we found that the clinical interventions, for example chemotherapy, can significantly improve the survival of LUAD patients harboring STK11 mutation (**Figure 3**). Here, we utilized the GDSC database for screening the sensitive and selective drugs for STK11 mutation LUAD. The screening results showed that Nutlin-3a(-) displayed the potential sensitivity and selectivity for LUAD with STK11 mutations (**Figure 4**). Our virtual screening provides one potential lead compound for LUAD with STK11 mutations.

As previously reported, STK11 plays an essential role in PI3K/RAS/mTORC1 signaling pathway and anticipates the various critical pathways, including the immune regulation (20). To well understand the biological functions associated with STK11 mutations in LUAD, we analyzed RNA-seq data on LUAD patients with and without STK11 mutations in the TCGA database and identified alterations in gene expression (**Figure 6A**). GSEA enrichment analysis (**Figure 5**) showed that the STK11 mutations were associated with multiple cancer-related pathways, including oxidative phosphorylation, unfolded protein response, peroxisome, protein secretion, Wnt/beta catenin signaling and PI3K/AKT/mTOR signaling. Oxidative phosphorylation is the metabolic approach, which provided the ATP for biological process. Owing to the critical role of STK11 in PI3K/RAS/mTORC1 signaling pathway, the mutation of STK11 caused the lower STK11 expression level. To consider the inhibition role of STK11/AMPK1 complex to mTOC1 protein, the lower STK11 expression will cause the continuous activation of mTOR signaling pathway (21). Moreover, the enriched mTOR signaling pathway indicated that STK11 mutation is positively associated with the worse survival of LUAD patients, because the activation of PI3K/AKT/mTOR signaling plays an essential role in promoting tumorigenesis, cancer development by various mechanisms, including those involving gene mutation, PTEN downregulation and activation of oncogene receptors (22–24).

Additionally, there were 746 DEGs between the STK11 mutation and wild-type groups (**Figure 6**). By performing the GO and KEGG enrichment of DEGs, the results showed that these DEGs can be classified into various annotations, *i.e.* extracellular matrix disassembly, cytokine activity, G protein-coupled receptor binding, receptor ligand activity, cytoplasmic vesicle lumen, arachidonic acid metabolism, phenylalanine metabolism, histidine metabolism and tyrosine metabolism. Owing to the role of STK11 in PI3K/RAS/mTORC1 signaling pathway, to silence the STK11 genes can further affect the membrane proteins, protein transcription and energy metabolic process. The GO and KEGG results confirmed that silencing of STK11 can regulate more active cellular signaling pathways and metabolic processes. This analysis reveals associations between STK11 mutations and cellular progression and offers insights into potential therapeutic mechanisms that could underlie individualized treatment of LUAD patients with STK11 mutations.

Based on the PPI network analysis (**Figure 7**), the top four modules in the PPI network were highly consistent with KEGG and GO analysis, which indicated enrichment regarding chemokine signaling pathway, neutrophil activation involved in immune response, neutrophil activation, receptor ligand activity, and protein digestion and absorption. This again shows that LUAD cases with STK11 mutations tend to exhibit high activity related to a variety of cellular processes, including immune infiltration signaling.

Moreover, we also found that there were 21 genes, more frequently mutated in the STK11 mutation group than the wild-type group (p-value < 0.01). The subsequent KEGG analysis results (**Figure 8**) revealed that these frequently mutated genes can be classified into FoxO signaling pathway, central carbon metabolism in cancer, NSCLC, ErbB signaling pathway, microRNAs in cancer and gap junction. These results are consistent with the KEGG and GO analysis results, which indicated that STK11 mutation can activate or inactivate numerous specific signaling pathways related with metabolic and immune response.

In conclusion, we evaluated the relevance of STK11 mutations in LUAD regarding immune cell infiltration, drug sensitivity and various cellular signaling pathways. The systematical investigation showed that the mutation of STK11 in LUAD can significantly affect the energy metabolic process and further downstream of immune infiltration. These enriched annotations reveal that the major signaling pathways associated with LUAD cases involving STK11 mutations. Moreover, we also identified one novel lead compound Nutlin-3a(-), which may provide the potential therapeutic candidate. Our investigation may help to improve the prediction of LUAD prognosis and personalize the treatment strategies used for these patients.

## Data Availability Statement

Publicly available datasets were analyzed in this study. This data can be found here: https://portal.gdc.cancer.gov/; https://www.cbioportal.org/;https://cistrome.shinyapps.io/timer/.

## Author Contributions

ZL conceived and designed the study, obtained funding, and drafted the manuscript. ZL and JX acquired the data and drafted the manuscript. PZ critically revised the manuscript. BD and KM performed statistical analysis and technical support. All authors contributed to the article and approved the submitted version.

## Conflict of Interest

The authors declare that the research was conducted in the absence of any commercial or financial relationships that could be construed as a potential conflict of interest.
